# Deep Learning‐Driven Computational Imaging for Noninvasive Monitoring System of Brain Temperature and Metabolism: A Hypothermia Validation for Acute Ischemic Stroke

**DOI:** 10.1002/mco2.70841

**Published:** 2026-08-05

**Authors:** Tianhang Yang, Qihan Zhang, Yilun Huang, Yuan Wang, Fuzhi Cao, Xin Zhang, Ming Wei, Qingfeng Ma, Hongzhi Kuai, Ming Li, Jianzhuo Yan, Miaowen Jiang, Xunming Ji

**Affiliations:** ^1^ School of Information Science and Technology Beijing University of Technology Beijing China; ^2^ Beijing Institute of Brain Disorders Capital Medical University Beijing China; ^3^ China‐America Institute of Neuroscience and Beijing Institute of Geriatrics Xuanwu Hospital Capital Medical University Beijing China; ^4^ Department of Neurology and Neurosurgery, Xuanwu Hospital Capital Medical University Beijing China; ^5^ School of Instrumentation and Optoelectronic Engineering Beihang University Beijing China; ^6^ School of Engineering Medicine Beihang University Beijing China; ^7^ Brainnetome Center, Laboratory of Brain Atlas and Brain‐inspired Intelligence, Institute of Automation Chinese Academy of Sciences Beijing China; ^8^ Tianjin Huanhu Hospital Tianjin University Tianjin China; ^9^ Graduate School of Engineering Maebashi Institute of Technology Maebashi Japan

**Keywords:** brain temperature imaging, deep learning, hemodynamics, hypothermia neuroprotection, metabolism thermodynamics

## Abstract

Brain temperature (BT) is a critical physiological indicator closely associated with neurological function and disease progression. However, real‐time, noninvasive monitoring of BT remains a challenge due to the limitations of current technologies. Here, we present a novel multimodal framework combining bioheat transfer modeling, deep learning, and computational thermography for accurate BT prediction and imaging. A one‐dimensional convolutional neural network was trained on multimodal clinical data, integrating cerebral blood flow, tissue oxygen saturation, and intracranial pressure, achieving a mean absolute error of 0.31°C in BT prediction. The framework incorporates finite element analysis to generate 3D thermographic maps of brain tissue with a spatial resolution of 0.4 mm, validated using MRI‐derived data. This approach demonstrated robust performance in predicting localized temperature variations in acute ischemic stroke patients undergoing therapeutic hypothermia, with deviations below 0.45°C. Our findings highlight the potential of this system to enable precise BT monitoring, bridging the gap between computational modeling and clinical neuro‐thermometry, and paving the way for advanced diagnostic and therapeutic interventions.

## Graphical abstract

This study presents the first proof‐of‐concept framework for 3D brain temperature and metabolism imaging using deep learning and clinical validation. An integrated multimodal instrument enables noninvasive neuro‐monitoring, while MRI‐based 3D modeling and computational imaging improve prediction accuracy, visualization quality, and spatial resolution. This approach provides a novel pathway for real‐time brain function assessment.

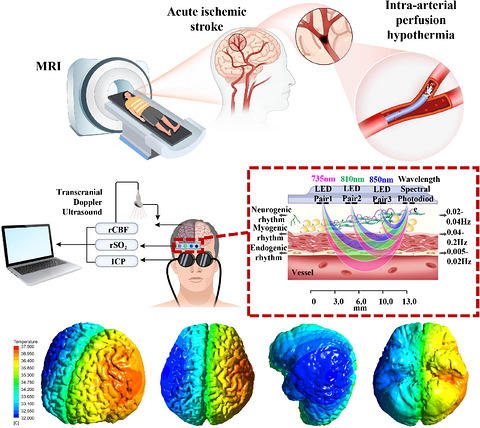

## 1 Introduction

Brain temperature (BT) is a critical physiological indicator reflecting the dynamic balance between heat production, cerebral blood flow (CBF), and metabolic activity [1]. Precise regulation of BT is essential for maintaining neuronal homeostasis, and its dysregulation has been implicated in adverse outcomes in neurological disorders [2, 3]. For example, elevated BT is closely associated with worsened ischemic brain injury and increased mortality, with a 1°C rise exacerbating tissue damage [4, 5]. Real‐time, noninvasive monitoring of BT remains challenge due to technological limitations [6, 7]. The lack of accurate real‐time BT monitoring has driven increasing interest in BT prediction for neurological disorders [8, 9]. BT plays a critical role in various therapeutic applications, including therapeutic hypothermia (TH) for hypoxic–ischemic encephalopathy [10], traumatic brain injury [11], ischemic [12], and hemorrhagic [13] stroke, laser‐induced interstitial thermal therapy [14] for brain tumors, and targeted temperature management [15] during critical neurological emergencies. These interventions rely heavily on the accurate assessment and precise prediction of BT.

However, unclear and unquantified heat transfer mechanisms hinder the integration of BT monitoring into clinical practice. Therefore, the underlying mechanisms driving variations in BT and associated changes require further investigated. Furthermore, current BT measurement techniques face significant limitations [16, 17]. As a result, predictive models based on the classic Pennes’ bio‐heat transfer equation have been developed in biophysics to describe heat transport within biological tissues [18, 19, 20, 21].

Current BT prediction models face several challenges that limit their accuracy and applicability: (1) lack of quantitative hemodynamics analysis and clinical correlative mechanisms. Research indicates that CBF directly influences BT and cerebral metabolic rate of oxygen (CMRO_2_). However, no quantitative analysis has integrated the coupling mechanism among “CBF‐BT‐CMRO_2_” [22, 23, 24]; (2) absence of precise spatial localization for BT. Existing simplified bioheat transfer models fail to adequately capture the spatial distribution of BT. Advanced spatial localization techniques, such as those guided by magnetic resonance imaging (MRI), are therefore necessary to enable precise BT predictions at various locations within brain tissue [17, 25, 26].

Deep learning (DL) enables automatic extraction of complex features through end‐to‐end learning [27, 28]. Consequently, models can learn rich patterns and underlying rules directly from the data, showcasing significant potential across various fields [29, 30]. Moreover, in DL, fine‐tuning [31] is a commonly used class of transfer learning techniques. Fine‐tuning adapts a pretrained model to a related target dataset. With this technique, the performance of the model on a specific task can be effectively improved. At the same time, the required training time as well as the amount of data can be significantly reduced. The fine‐tuning technique has demonstrated a strong potential for clinical applications [32, 33, 34], especially when faced with insufficient data or limited computational resources. In this study, coupling mathematical models integrating CBF, BT, region cerebral tissue oxygen saturation (rSO_2_), and intracranial pressure (ICP) were developed based on DL methodologies. Unlike previous studies [23, 35, 36], ICP was incorporated into the BT model as a hemodynamic parameter. This integration not only accounts for the intrinsic relationships between these variables but also leverages domain‐specific knowledge to tailor the model for specific clinical contexts, thereby establishing a refined and densely mapped prediction framework.

Furthermore, an integrated instrument system was designed for multimodal, noninvasive neuro‐monitoring of brain function. The system collected and analyzed ICP, region CBF (rCBF), and rSO_2_ from the prefrontal cortex in 50 healthy individuals, using temperature measurements that substituted BT. A dataset of 100 measurements was obtained. By employing a 1D‐convolutional neural network (1D‐CNN) that integrates domain knowledge, we achieved a more accurate and comprehensive prediction of BT. The limitations of localized noninvasive monitoring were addressed using a three‐dimensional (3D) finite element analysis (FEA) model based on bioheat transfer theory, enabling the calculation of whole brain BT distribution. Using MRI data from three acute ischemic stroke (AIS) patients undergoing intravascular hypothermia therapy, as described in a previous study [37], the FEA model calculated BT values for each voxel of the entire brain and specifically delineated regions of ischemic infarction. These BT values were overlaid onto corresponding e MRI brain image voxels, resulting in high‐resolution spatial maps that highlighting areas of higher or lower temperatures, effectively bridging the gap between mathematical modeling and brain thermography. These findings demonstrate that high spatial resolution for BT can be achieved by finely discretizing the cranial model into small grid elements and innovatively constructing a map of BT and metabolic thermodynamics. Therefore, the proposed BT instrument system, which integrates neuro‐monitoring, DL modeling, and computational imaging techniques, holds significant potential for improving safety and efficacy during clinical therapeutic procedures. This research contributes to the development of brain thermometry and the development of wearable devices, facilitating the integration of BT into clinical decision‐making and further bridging the gap between mathematical modeling and brain thermography.

## 2 Results

### 2.1 Multimodal Noninvasive Neuromonitoring System

**FIGURE 1:**
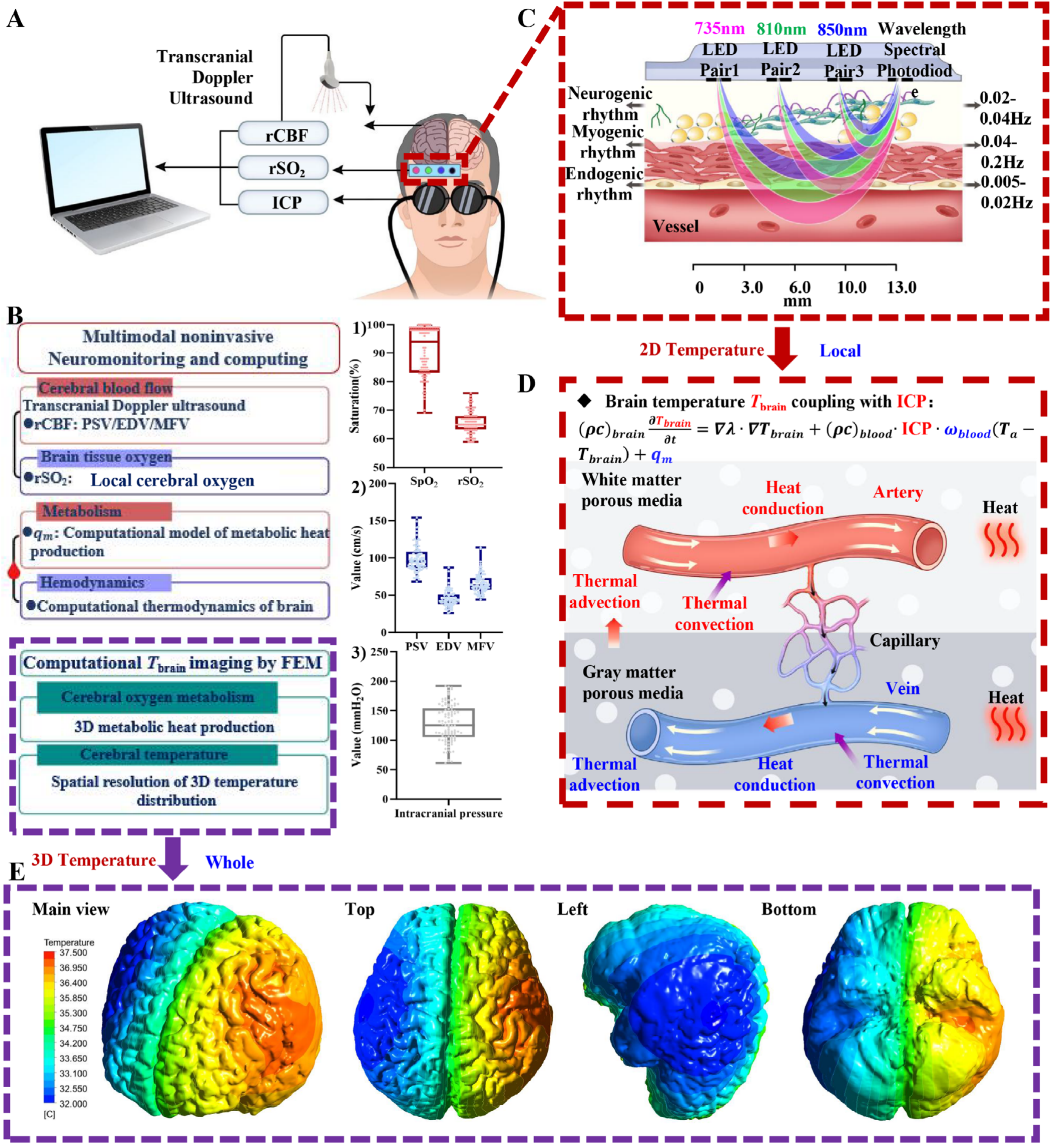
Overview of noninvasive neuromonitoring. (A) The multimodal noninvasive neuromonitoring system. (B) Measurements include rSO_2_–S_p_O_2_ (1), rCBF (2), and ICP (3), combined computational heat transfer methods for thermodynamic analysis and “brain temperature–cerebral oxygen metabolism” three‐dimensional (3D) imaging. (C) A schematic diagram illustrating the principle of brain oxygen measurement, highlighting the light path based on wavelength and distance for optical measurements of the prefrontal tissue (the skull was excluded, focusing solely on vascular tissue). (D) A rigorous biophysical model for the prediction brain temperature, defining three heat transfer domains (arteries, veins, and tissue) and three modes of energy transfer (conduction, convection, and advection). (E) The FEM (finite element method) heat transfer analysis method applied to a 3D brain tissue model, visualized from three directional views. *Abbreviations*: rSO_2_, regional cerebral oxygen saturation; S_p_O_2_, peripheral oxygen saturation; rCBF, regional cerebral blood flow; ICP, intracranial pressure; peak systolic velocity (PSV) refers to the maximum velocity reached by blood flow through a specific segment of a blood vessel during cardiac systole, reflecting the vessel's blood supply capacity and the presence of any stenosis. End‐diastolic velocity (EDV) refers to the blood flow velocity at the moment just before the end of cardiac diastole, reflecting peripheral resistance and perfusion in the distal part of the vessel. Mean flow velocity (MFV) refers to the time‐averaged blood flow velocity over a cardiac cycle, used to comprehensively assess overall blood flow.

A multimodal noninvasive neuromonitoring system was developed integrating near‐infrared optical instruments for brain oxygen saturation measurement, transcranial Doppler for CBF detection, and flash visual evoked potentials for ICP monitoring (Figure 1A). The system monitored rCBF and rSO_2_ in the prefrontal cortex of 50 healthy individuals, resulting in 100 data instances. In healthy individuals, rSO2 ranged from 60 to 70%, approximately 20–30% lower than peripheral oxygen saturation measured by pulse oximetry (S_p_O_2_: 85–98%). Measurements of rCBF included the peak systolic velocity (PSV: 98.75 ± 17.18 cm/s), end‐diastolic velocity (EDV: 44.94 ± 10.60 cm/s), and mean flow velocity (MFV: 65.97 ± 12.89 cm/s) (Figure 1B). Unlike previous studies, ICP was introduced into the BT model as a hemodynamic parameter. CP values (128.06 ± 31.78 mm H_2_O) were fitted as influencing factors (Figure 1B).

The anatomical structures and their respective rhythmic frequencies within the vasculature, surrounded by three anatomical components, facilitate endogenous spontaneous intrinsic oscillations [38] at 0.005–0.02 Hz (myogenic), 0.02–0.04 Hz (neurogenic), and 0.04–0.2 Hz (myogenic rhythms) [39]. For this analysis, the skull was excluded, with a focus solely on vascular tissue. A broadband LED combined with a multispectral photodiode enables simultaneous measurements at three wavelengths (735, 810, and 835 nm) for rSO_2_ monitoring. The principle underlying the rSO_2_ monitoring technique has been described in our previous research [40] (Figure 1C).

A whole‐BT calculation imaging algorithm was combined with DL to further enhance the accuracy of BT prediction, thereby addressing the limitations of localized noninvasive monitoring. BT originates from metabolic heat within brain tissue and vasculature (Figure 1D) and is transferred to surrounding tissues, modeled as a porous medium [41], via conduction, advection, and convection through the vascular network [17]. Energy and mass conservation were maintained locally and globally across the brain in each domain and were described using a set of governing equations. Notably, heat generated by metabolism plays a significant role in influencing changes in BT.

For the FEA heat transfer method based on the 3D brain tissue model, a mapping relationship was established using linear interpolation of temperature values to generate temperature maps. These images provided detailed views of BT and cerebral metabolic heat production from different perspectives. (Figure 1E)

### 2.2 DL‐Based Clinical Parameter Prediction Framework and Results

**FIGURE 2:**
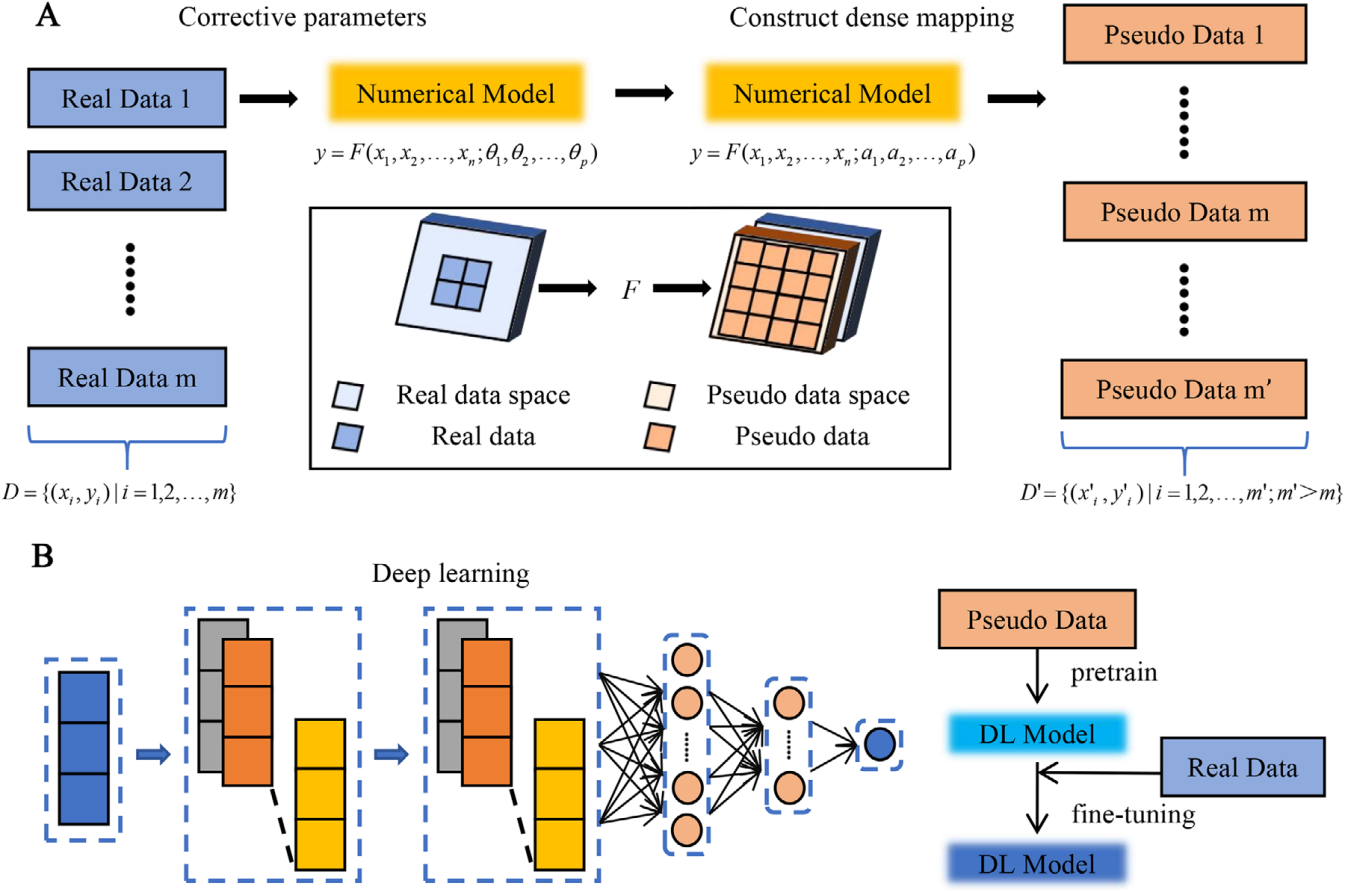
Overview of the proposed deep learning framework. (A) Deep learning framework incorporating domain knowledge. A dense mapping between features was constructed using limited real data combined with domain knowledge. Corrective parameters and numerical models were utilized to transform real data into a pseudo‐data space. This allowed for the prediction of clinically critical parameters by enriching the dataset with pseudo‐data, expanding the available training data. (B) Deep learning network architecture and training methodology. A deep convolutional neural network (CNN) was employed to extract and predict features, integrate domain knowledge, and adapt to clinical data. The training process involved two steps: pretraining the model on pseudo‐data followed by fine‐tuning using real clinical data to optimize performance for practical applications.

DL‐based clinical parameter prediction framework (BT‐DL algorithm) addresses sparse parameter mapping caused by limited samples and predicts physiological features. Furthermore, the framework effectively mitigated the theoretical errors inherent in traditional analytical methods [42]. In this study, the method established a dense mapping between features, encompassing a wide range of data distributions (including both normal and abnormal values from patients and healthy individuals), enabling further fine‐tuning of the model (Figure 2).

**FIGURE 3:**
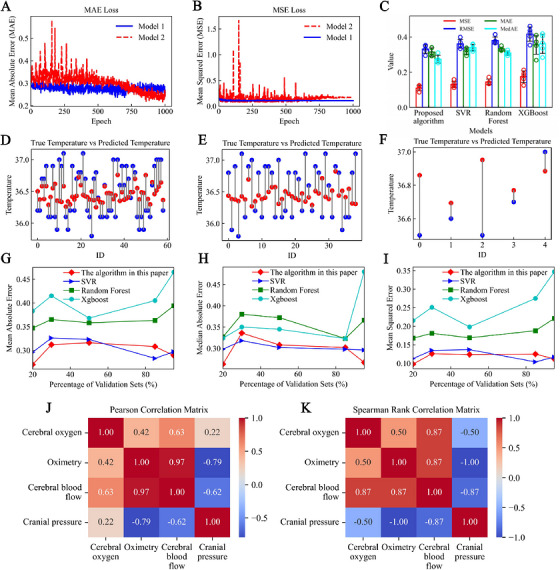
Comparative performance of deep learning framework and other artificial intelligence (AI) algorithms. (A and B) Performance comparison between the deep learning model trained using the proposed method and the model trained solely on clinical data, evaluated on the training and validation sets. (C) Comparative performance of various AI algorithms on the validation set, red dots represent predicted values, blue dots represent actual values. (D–F) Error analysis showing the difference between predicted and actual values on the training set, validation set, and test set using the proposed method. (G–I) Performance comparisons of AI algorithms when training and validation sets are split at different percentages. (J and K) Correlation analysis between intracranial pressure and other physiological features.

To evaluate the contribution of the proposed training strategy, an ablation experiment was conducted. The deep neural network (DNN) was fine‐tuned using real sample data to establish a BT prediction model. Coupling rSO_2_–S_p_O_2_, ICP, and rCBF were selected as input features, where ICP and rCBF were as perfusion terms for cerebral hemodynamic [43], and BT was set as the output. The dataset consisted of 100 samples, which were divided into nonoverlapping training (60%) and validation sets (40%). The effectiveness of the proposed method was evaluated by comparing the errors in the training and validation sets between the BT‐DL algorithm and a DNN trained solely on small‐sample clinical data. The training and validation errors of the DNN under two different training methods are shown in Figure 3A,B. (Model 1 and Model 2 represent the proposed method and the conventional training method, respectively.) The mean squared error (MSE) loss values for Model 1 were 0.08 on the training set and 0.11 on the validation set.

In this study, several widely used AI algorithms, including Random Forest (RF), eXtreme Gradient Boosting (XGBoost), Support Vector Regression (SVR), and the BT‐DL algorithm, were employed to predict BT characteristics based on multiple clinical features. The performance of these algorithms evaluated using prediction error metrics, specifically MSE, root MSE (RMSE), mean absolute error (MAE), and median absolute error (Med‐AE) (Figure 3C). All models were trained and evaluated using fivefold cross‐validation on the same dataset.

Paired two‐tailed *t*‐tests were performed on cross‐validation results to assess statistical differences. The RF algorithm achieved MSE, RMSE, MAE, and Med‐AE values of 0.14, 0.38, 0.33, and 0.30, respectively, on the validation set, indicating its potential for clinical applications despite the limited clinical case data. Although XGBoost showed slightly higher overall sample error compared with the RF, it demonstrated competitiveness with MSE, RMSE, MAE, and Med‐AE values of 0.17, 0.41, 0.35, and 0.36, respectively. SVR, used as one of the comparative baselines, exhibited performance between RF and XGBoost, achieving MSE, RMSE, MAE, and Med‐AE values of 0.13, 0.36, 0.32, and 0.34, respectively. The BT‐DL algorithm showed a numerical advantage over the compared algorithms, with MSE, RMSE, MAE, and Med‐AE values of 0.11, 0.34, 0.31, and 0.27, respectively. These results demonstrate the superior accuracy of the BT‐DL algorithm under limited data. Furthermore, to statistically validate the performance differences between algorithms, we calculated the coefficient of determination (*R* ^2^) for each method based on fivefold cross‐validation results. Overall, the BT‐DL model demonstrated a higher *R* ^2^ value (0.25) compared with other methods, while RF (0.16), XGBoost (0.15), and SVR (0.20) exhibited relatively lower coefficients. Subsequently, paired *t*‐tests conducted based on the performance of each fold revealed that the *p* values between algorithms were all greater than 0.3. This indicates that, under the current conditions of small sample size, although consistent performance trends were observed between models, the statistical significance was not pronounced. This phenomenon is primarily attributed to the limited number of clinically available samples.

The clinical feasibility of the proposed method was further evaluated using clinical samples. The output values of the DNN trained with the BT‐DL algorithm were inverse‐normalized into predicted temperature value. The errors between the predicted and actual measured temperature values on the training and validation sets are shown in Figure 3D,E. During the training phase, the BT‐DL algorithm demonstrated excellent performance in absolute error metrics, with a median, mean, maximum, and minimum of 0.31, 0.32, 0.70, and 0.01°C, respectively. These results indicate that the BT‐DL algorithm converged effectively during training. Similarly, the model exhibited robust performance on the validation set, achieving a median error, mean, maximum, and minimum error of 0.36, 0.4, 0.79, and 0.07°C, respectively. These findings confirm that the model learned deeper mapping rules between feature parameters and BT rather than merely memorizing the training samples, further validating the BT‐DL algorithm's generalization capability on unseen data. Overall, the BT‐DL framework effectively detected intrinsic patterns between clinical features and BT, even with limited clinical data. The BT‐DL algorithm demonstrated stability and accuracy in its predictive capability. The noninvasive BT prediction system was tested on samples from five clinical patients to compare its comparisons with actual BT measurements and evaluate its performance in real medical scenarios. The deviations between the predicted values and the actual BT observations, measured as absolute errors were 0.36, 0.09, 0.45, 0.06, and 0.11°C, with the maximum deviation capped at 0.45°C (Figure 3F). These results demonstrate the stability of the system's prediction accuracy and reflect a tight distribution of prediction errors, further validating its reliability and accuracy. To further assess the robustness of the BT‐DL model, we conducted supplementary analyses on the expanded test sample comprising 10 AIS patients while maintaining the original training dataset. Five additional independent clinical cases were incorporated into the test set, and the model was retrained under identical training configurations. Results indicate that the error distribution broadly aligns with the original five‐case test outcomes, demonstrating stable predictive performance. Detailed results are presented in Figure S1.

### 2.3 The BT‐DL Algorithm Performance Under Different Data Capacities

The performance of various AI algorithms was also compared under different training and validation set splits (training set‐validation set: 80–20, 50–50, 30–70, 15–85, 5–95%) using a dataset of 98 cases (Figure 3G–I). Overall, MAE and MSE curves for the XG‐boost and RF algorithms showed an upward trend when the training set size did not exceed 50%, as the proportion of validation set data increased. Notably, the Med‐AE curve rose sharply when only 5% of the data were used for training, indicating a pronounced overfitting state, and resulting in poorer prediction performance on the validation set. The SVR algorithm demonstrated competitiveness with the BT‐DL algorithm on small datasets. Specifically, the three curves’ metrics (MAE, MSE, and Med‐AE) remained relatively stable as the proportion of training data increased, indicating good generalization and stability on the validation set for both algorithms. However, the BT‐DL algorithm consistently achieved lower errors compared with SVR and the other algorithms, particularly when the validation set constituted 20% of the data. Furthermore, the BT‐DL algorithm exhibited superior performance under extreme conditions, with lower errors even when the validation set contained for 95% of the data.

### 2.4 Correlation Analysis of Patients' physiological Characteristics

The Pearson and Spearman correlation coefficient matrices for the rSO_2_–S_p_O_2_, BT, and rCBF were calculated to analyze the impact of ICP on other features. Pearson correlation measures linear relationships between variables. Values of +1, −1, and 0 represent perfect positive correlation, perfect negative correlation, and no linear relationship, respectively. The Spearman rank correlation coefficient measures the strength and direction of a monotonic relationship between two variables, including nonlinear trends, with values ranging from −1 to +1. The correlation coefficient between ICP and S_p_O_2_ was 0.22, indicating a slight positive correlation. Between ICP and rSO_2_, it was −0.79, indicating a strong negative correlation, and between ICP and rCBF was −0.62, also indicating a strong negative correlation (Figure 3J). Similarly, the Spearman correlation coefficients showed a moderate negative correlation (−0.5) between ICP and S_p_O_2_, a very strong negative correlation (−1) between ICP and rSO_2_, and a strong negative correlation (−0.82) between ICP and rCBF (Figure 3K). Overall, ICP exhibited a strong negative correlation with S_p_O_2_ and rCBF, suggesting that both S_p_O_2_ and rCBF may decrease as ICP increases, and vice versa. Notably, the trends between ICP and rSO_2_ differed between the two correlation coefficients, likely because the Pearson correlation focuses on linear relationships, while the Spearman correlation captures nonlinear monotonic trends. The Spearman correlation suggested that rSO_2_ might decrease as ICP increasing.

### 2.5 Modeling and Imaging of BT and Metabolism

**FIGURE 4:**
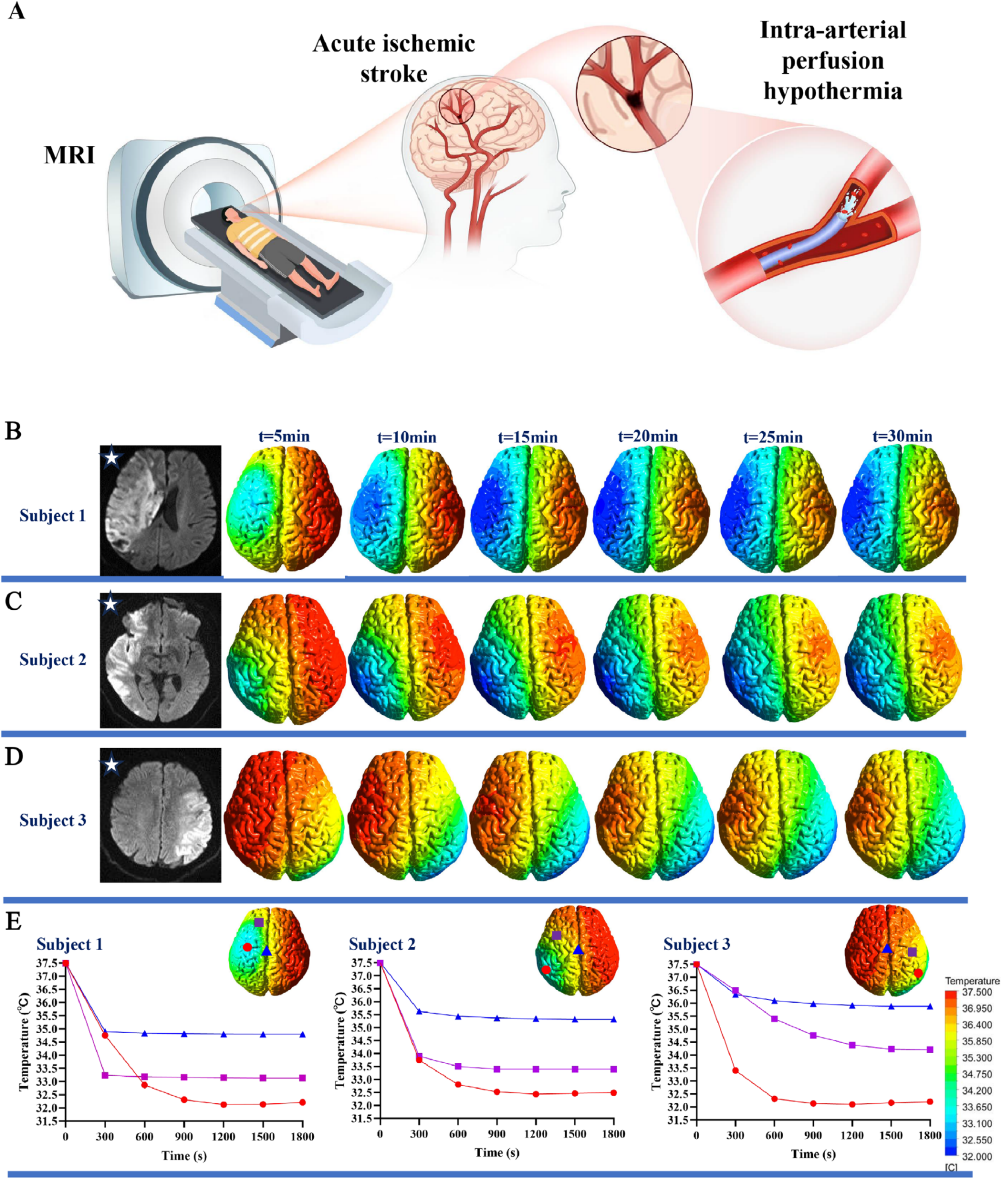
Three‐dimensional (3D) computational brain temperature imaging for acute ischemic stroke (AIS) patients using magnetic resonance imaging (MRI). (A) The process of 3D brain model construction based the MRI data; (B–D) brain temperature imaging results based on finite element analysis (FEA) at different infarct volume influence coefficients during a 30‐min hypothermia treatment for three AIS patients (indicated by “★”) [37]; (E) brain temperature curve at selected points: the central point of the brain tissue (blue) and two points within the targeted low‐temperature region (red and purple).

We took the BT in the targeted hypothermia region of the above DL enhanced bioheat transfer model as the initial values and realized the distribution and change trend of BT with gridded spatial resolution through 3D‐FEM analysis method. The retrospective study involving three patients with AIS related to middle cerebral artery (MCA) occlusion, who received short‐duration intravascular hypothermia therapy combined with mechanical thrombectomy, was conducted for further analysis, that details of the hypothermia therapy have been previously described [37] (Figure 4A). In our previous study [44], through the hemodynamic calculation and analysis based on the real blood vessel network model of human body, the exit temperature of the hypothermia interventional catheter can be assumed to be 31.2–32.0°C.

The whole‐brain 3D‐BT map within the infarct regions were generated using MRI‐based image segmentation to delineate the spatial extent of cerebral infarction. Processed MRI images were enhanced to a higher resolution and recolored to emphasize key details, ensuring an equitable representation of skull, white matter, gray matter, and cerebrospinal fluid. Also, erroneous images were corrected or discarded to maintain accuracy. The 3D‐BT distribution during whole‐brain tissue cooling was mapped using FEA‐based computational heat transfer modeling. As shown in the Figure 4B–D, the targeted hypothermia zone, including the cerebral hemisphere affected by infarction, was maintained for 30 min to achieve moderate hypothermia in the subjects. The 3D‐BT in the nonperfused brain regions was kept between 34.5 and 37.5°C during the 30‐min cooling phase. Temperature measurements were taken at the central brain tissue point (blue) and two points within the hypothermia target zone (red and purple represented the infarct core area and ischemic penumbra area, respectively) (Figure 4E).

For subject 1, the central brain tissue experienced a temperature drop of 2.5°C, while the infarct core area stabilized at around 32.5°C, and the ischemic penumbra area dropped to about 33.0°C. The infarct core area of the subject 2 showed little difference with subject 1, while the temperature in the central brain and ischemic penumbra tissue increased by 0.5°C than subject 1. The results showed that even if the initial hypothermia BT values were approximately the same, different infarct locations would lead to different degrees of hypothermia temperature. For subject 3, the BT in the central brain tissue increased by 1.5°C, with about 3.0 and 5.0°C decrease at the infarct core area and ischemic penumbra tissue, respectively. For the example of subject 1, the BT in the infarct core area was stabilized at around 32.8 ± 0.29°C with no spatial properties at 30 min from deep‐learning prediction, slightly higher than the 3D‐BT computational imaging result (32.5°C at 30 min in the infarct core area).

**FIGURE 5:**
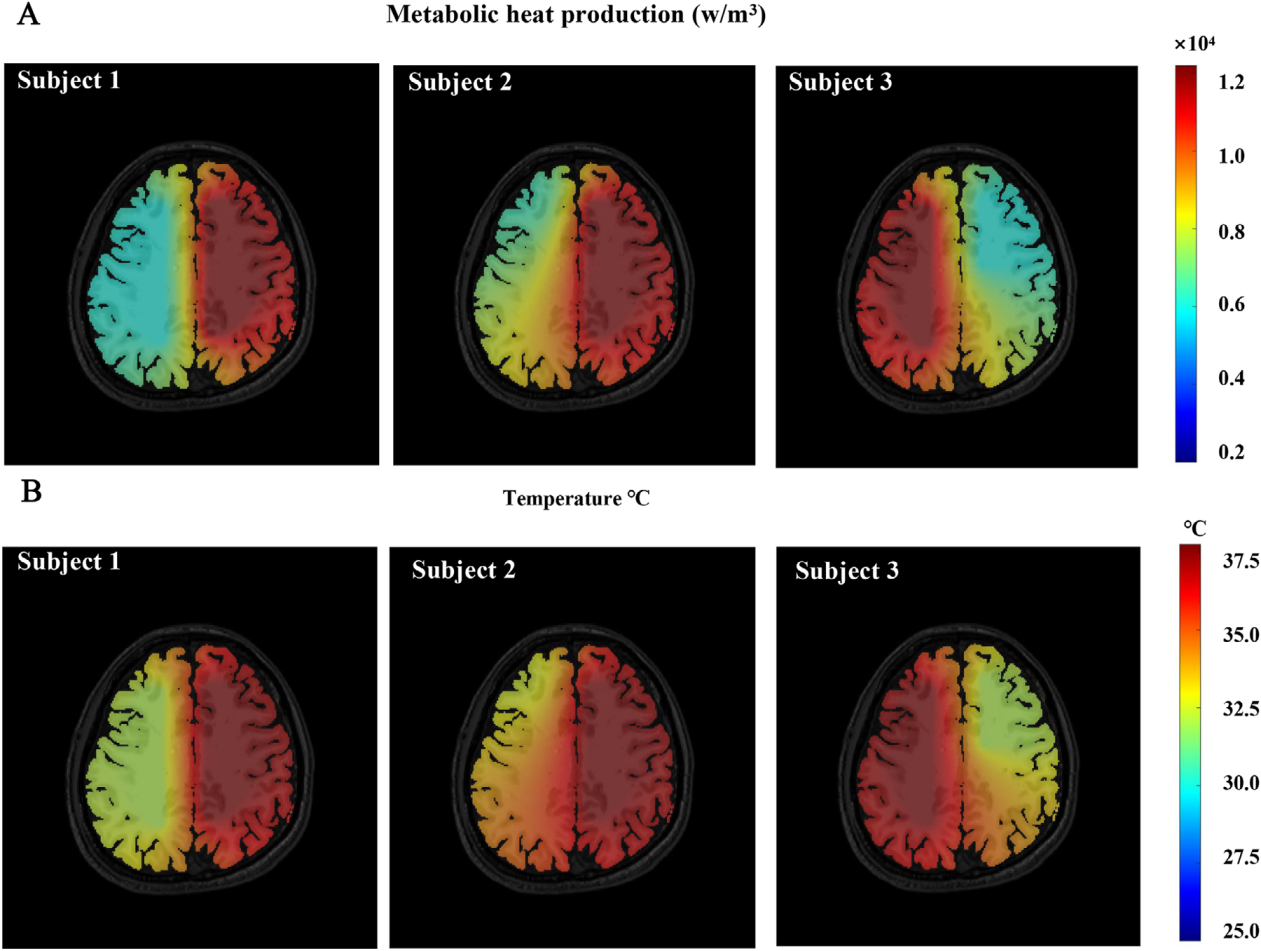
Model‐predicted metabolic heat production and brain temperature maps for three acute ischemic stroke (AIS) patients treated with intravascular hypothermia therapy. (A) Metabolic heat production maps. (B) Brain temperature maps.

A 3D spatial image of BT and metabolic heat production was constructed using original MRI images and BT prediction model (Figure 5). The MRI‐derived imaging depicted metabolic thermogenesis across the entire brain and in localized spatial regions using linear interpolation temperature mapping. In the perfused brain regions (the infarct area), the metabolic heat production (Figure 5A) was approximately 0.6–0.8 × 10^4^ W/m^3^, and the temperature ranged from 30 to 33°C during the 10‐min cooling period (Figure 5B). In the nonperfused brain regions (healthy area), the metabolic heat production increased to 1.0–1.2 × 10^4^ W/m^3^, and the temperature ranged from approximately 34.5–37.5°C. The tympanic membrane temperature in AIS patients treated with hypothermia decreased by approximately 2°C, which was slightly below the cooling range predicted by the computational model.

## 3 Discussion

The BT instrument system integrates neuro‐monitoring, DL, and computational imaging to improve safety and efficacy in clinical procedures. This research facilitates the development of advanced brain thermometry and wearable devices, enabling the integration of BT into clinical decision‐making and bridging the gap between mathematical modeling and brain thermography.

The BT prediction model provides a theoretical and methodological basis for accurately measuring BT, a critical parameter for optimizing clinical treatment [45]. As shown in Figure 1D, the model integrates three frameworks [46]: (1) uniform medium approximation, assuming thermal equilibrium between tissue and capillaries; (2) vascular structure modeling, capturing energy transfer (conduction, advection, and convection) across discrete domains via control volume theory; and (3) porous medium integration, simulating blood flow through voxelized tissue. Table S1 summarizes key clinical applications and findings from the past two decades.

Current theoretical analytical methods lack precision and a unified framework. The discrepancy between theoretical estimates and actual measurements may reflect implicit relationships that bring predictions closer to true values [43], but these relationships are patient‐specific and influenced by disease conditions and other factors, which may introduce theoretical errors [47, 48]. In this study, theoretical derivations, as domain knowledge, were integrated with DL models to enhance the predictive performance of BT prediction models.

DNNs were employed for complex mapping due to two advantages: (1) superior representational power over classical algorithms in fitting complex nonlinear relationships [43, 49]; and (2) a hierarchical architecture that extracts abstract features, significantly improving model robustness and tolerance to input variations.

In the BT‐DL algorithm, Model 1 exhibited significantly faster convergence than Model 2 on the training set. This is primarily attributed to the patient case data generated from domain knowledge, which encompassed various relationships among rSO_2_, S_p_O_2_, CBF, ICP, and BT. These mapping patterns provided Model 1 with superior initial parameters, allowing it to adapt rapidly to clinical cases by leveraging shared relationships between domain knowledge and real‐world data. The loss error of Model 2 steadily decreased after 750 epochs during the DNNs. However, the loss error on the validation set exhibited an overall upward trend, indicating that Model 2 overfitted the limited patient case data. The strong learning capability of the BT‐DL algorithm caused the model to memorize specific patient data in the training set rather than learn the mapping patterns between patient characteristics and BT, primarily due to the limited data volume. Overfitting reduced its generalization ability and increased prediction errors on unseen parameters. Model 1 outperformed Model 2 on unseen data, as evidenced by the loss error on the validation set, indicating stronger generalization capabilities and more accurate BT predictions for unknown patient cases. Additionally, Model 1 converged significantly faster than Model 2 on the validation set and showed no signs of overfitting. This confirms that Model 1 effectively generalized domain‐derived knowledge to real clinical data, successfully addressing the challenge of data sparsity in clinical domains.

DL models uncover implicit relationships in data. Moreover, the robustness of DL models mitigates the impact of noise and enhances their generalization abilities.

Above all, the noninvasive BT prediction system achieved superior performance despite clinical data scarcity. The BT‐DL algorithm, incorporating domain expertise, was developed using a 1D‐CNN to learn intrinsic patterns from limited clinical data. Five patients with AIS involving the MCA were evaluated to verify the algorithm. The BT‐DL algorithm successfully prediction BT from healthy individuals to AIS patients, integrating the coupling theory of bioheat transfer to simulate real clinical scenarios while maintaining its validity. This approach provides a theoretical foundation and scientific hypothesis for 3D‐BT imaging with spatial resolution. The FEM‐3D BT model was also validated using clinical data from intracranial cooling and direct BT measurements.

The model holds significant potential for enhanced generalization and performance improvement as additional patient data become available. Despite data scarcity, this noninvasive system demonstrated high accuracy and practicality, offering significant potential to enhance patient care and personalized treatment in neuroscience and clinical medicine.

To address the common challenge of clinical data scarcity [50], we adopted active data accumulation and continuous patient enrollment. We pretrained our neural network on a large synthetic dataset to ensure robust feature representation, followed by iterative optimization to achieve the high clinical reliability necessary for improving treatment quality.

Unlike directly training a neural network with the entire dataset, our approach mitigates overfitting caused by the limited sample size, which fails to represent the distribution of the overall data space. Fine‐tuning mitigates the need for large datasets, which are often scarce in clinical settings [51]. The DNN is pretrained on a synthetic dataset derived from domain knowledge [52], mapping rSO_2_, S_p_O_2_, CBF, ICP, and BT, to learn rich feature representations. Subsequent fine‐tuning of the output layer weights [53] then adapts the broad feature distributions captured during pretraining [54] to specific clinical diagnostics, effectively overcoming the challenge of sample scarcity.

This study employs continuous data acquisition and iterative optimization. By establishing a data‐sharing mechanism with Xuanwu Hospital, the team collects new patient data quarterly to retrain and validate the model. This establishes a cyclical optimization process encompassing data accumulation, model pretraining, fine‐tuning, performance evaluation, and redeployment, thereby progressively enhancing the model's clinical adaptability and generalization capabilities.

While our system advances noninvasive BT monitoring, clinical implementation entails technological hurdles and resource requirements. Future research will focus on advancing portable monitoring solutions and refining real‐time analytics for enhanced clinical utility.

Due to medical ethics and surgical limitations, this study did not perform real‐time monitoring during intravascular hypothermia. Instead, the DL prediction model and AIS patients’ validation method were used to approximate the relationship between neural function and BT in AIS patients. The predicted BT value from the DL model was feasibly assumed as the targeted cooling temperature for AIS patients undergoing hypothermia therapy. This method provides a theoretical basis for future clinical guidelines. Additionally, when five AIS patients were selected as the validation set, slight changes in BT caused by mild brain injury were disregarded, and body temperature was used as a substitute for BT.

## 4 Conclusion

The 3D BT prediction model has garnered significant attention in various therapeutic approaches for neurological disorders, including TH for hypoxic–ischemic encephalopathy, hypothermic neuroprotective therapy for ischemic and hemorrhagic stroke, laser‐induced thermotherapy for targeted brain tumor ablation, and applications in neurocritical care. BT prediction models serve several key functions in clinical practice: (1) analyzing heat transfer within brain tissue, mechanisms of temperature regulation, and influencing factors; (2) providing theoretical guidance and methodological support for accurate BT measurements; (3) acting as a critical parameter to guide and optimize clinical treatment strategies.

Overall, the BT‐DL architecture was developed to addresses the challenge of sparse mappings between patient features and key physiological parameters caused by data scarcity, enabling its application in clinical practice. In addition, an efficient noninvasive BT prediction system was developed for practical medical scenarios by integrating real‐time monitoring capabilities of precision medical instruments, potentially enhancing the accuracy of diagnosis and treatment. The proposed framework represents significant progress in addressing medical prediction challenges through advanced artificial intelligence technologies in data‐limited environments.

## 5 Materials and Methods

### 5.1 DL‐Based Model for BT Prediction

As illustrated in Figure 2A,B, a DL framework integrating domain knowledge was developed to predict critical clinical parameters, addressing the sparse mapping challenge in limited‐data clinical scenarios. This approach overcomes theoretical inaccuracies of traditional analytical methods. The framework is not limited to specific parameters, and various deep network architectures can perform similarly well.

Fifty healthy subjects were recruited for this study, and five AIS patients (caused by occlusion of the MCA) aged 18 years or older. Patients with cerebral edema, hemorrhage, tumors, or infections were excluded for thermal modeling homogeneity. Ambient temperature was rigorously controlled to minimize external variability. All subjects provided written informed consent prior to participation. The healthy subjects were evaluated based on four key parameters: brain oxygenation, peripheral oxygen saturation, ICP, and CBF (measured using transcranial Doppler). Each parameter was measured twice for each subject, resulting in 100 datasets for training and validation. For the test set, body temperature, brain oxygenation, and MRI (T1‐weighted and diffusion‐weighted imaging) data were collected. Subjects were positioned supine after resting quietly for at least 5 min before assessments. This study was approved by the Ethics Committee of Xuanwu Hospital, Capital Medical University (approval number: 2021–053). The protocol for intravascular hypothermia therapy has been previously described [37].

The following methods are used to build domain knowledge. Van't Hoff's equation [55] has been widely applied in both human and animal studies to describe the relationship between temperature and the cerebral oxygen metabolic rate (rCMRO_2_), which is closely linked to rSO_2_. In this study, a multimodal analysis model analyzing “CBF–CMRO_2_–BT” coupling was developed based on our preliminary research findings [37]. The brain tissue temperature values, which were used to generate pretraining data, were calculated using the following equation:

(1) $$\ln\left(\frac{C_{a}O_{2}\cdot \left(S_{p}O_{2}-SO_{2}\right)}{\gamma \cdot S_{a}O_{2}}\cdot q_{0}\cdot 3^{\frac{\left(T-37\right)}{10}}\right)=a\left(\frac{1}{T}\right)+b$$

Here, CMRO_2_ was determined using the Fick principle [56]:

(2) $$\mathrm{CMR}\;O_{2}=C_{a}\;O_{2}\cdot \mathrm{OEF}\cdot \mathrm{CBF}$$

where OEF (oxygen extraction fraction) is defined as:

(3) $$\mathrm{OEF}=\frac{S_{p}O_{2}-\mathrm{rS}O_{2}}{\gamma \cdot S_{p}O_{2}}\;$$

and arterial oxygen content (C_a_O_2_) is given by:

(4) $$C_{a}\;O_{2}=\frac{1.36\mathrm{mL}\;O_{2}}{1\mathrm{gHgb}}\;\cdot \mathrm{Hct}\left(\%\right)\cdot \mathrm{MCHC}\left(\frac{\mathrm{gHgb}}{\mathrm{mL}\;\mathrm{blood}}\right)\cdot S_{p}O_{2}\left(\%\right)$$

C_a_O_2_ and OEF represent the arterial blood concentration of oxygen and the oxygen extraction fraction, respectively. The mean corpuscular hemoglobin concentration (MCHC) and mammalian hemoglobin oxygen binding capacity were set to 32–36 g/mL and 1.36 mL O_2_/g (Hgb), respectively [57]. ICP is closely associated with cerebral hemodynamics [36], and CBF varies with temperature according to the following relationship [23]:

(5) $$\mathrm{ICP}\propto q=q_{0}\cdot 3^{\frac{\left(T-37\right)}{10}}$$

These theoretical derivations established the interdependence among rSO_2_, S_p_O_2_, CBF, and BT. The DL framework enabled mappings between BT values and varying levels of rSO_2_, S_p_O_2_, CBF, and ICP by encoding this domain knowledge as data pairs.

Follow these steps to generate domain knowledge data. Initially, the values of *a* and *b* within the described mathematical model were calculated (Equation 1). Due to the difficulty of obtaining explicit solutions, the least squares method was employed for approximation. The equations were reformulated as follows:

(6) $$y_{i}=a\cdot \frac{1}{T_{i}}+b$$

To construct the objective function *E* (*a*, *b*), the partial derivatives of *E* with respect to *a* and *b* were set to zero. By minimizing *E* (*a*, *b*), the optimal values of *a* and *b* were calculated as follows:

(7) $$E\;\left(a,b\right)=\sum_{i=1}^{n}{\left(y_{i}-\left(a\cdot \frac{1}{T_{i}}+b\right)\right)}^{2}\;$$

(8) $$\frac{\partial E}{\partial a}=-2\;\sum_{i=1}^{n}\frac{y_{i}-\left(a\cdot \frac{1}{T_{i}}+b\right)}{T_{i}}=0$$

(9) $$\frac{\partial E}{\partial b}=-2\;\sum_{i=1}^{n}\left(y_{i}-\left(a\cdot \frac{1}{T_{i}}+b\right)\right)=0$$

To account for patient heterogeneity, data from 10 representative cases were selected. Once the values of a and b, the Newton‐Raphson method was utilized to initialize appropriate starting values (*T*_0 _= 36.5°C) and iteratively refine the estimate of brain tissue temperature *T* by solving *f*(*T*) = 0. The equation for *f*(*T*) was expressed as follows:

(10) $$f\;\left(T\right)=\ln\left(C_{a}O_{2}\cdot \frac{\left(S_{a}O_{2}-S_{t}O_{2}\right)}{\gamma \cdot S_{a}O_{2}}\cdot q_{0}\cdot 3^{\left(\frac{T-37}{10}\right)}\right)\;-a\left(\frac{1}{T}\right)-b$$

The iterative steps were as follows:

Step 1: calculate the first‐order derivative at the current iteration point $T_{n}$:

(11)

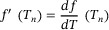

Step 2: update the point $\;T_{n}$:

(12)

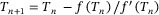

Steps 1 and 2 were repeated until convergence was achieved or the maximum number of iterations was reached. Consequently, BT values were derived for different levels of rSO_2_, S_p_O_2_, CBF, and ICP. A large dataset mapping these parameters was generated for pretraining the DL model.

To mitigate scale‐induced training bias among rSO_2_, S_p_O_2_, and CBF, data standardization was performed. As all measurements originate from clinically calibrated instruments, the dataset contains virtually no outliers. Consequently, we employed min–max normalization to enhance training stability. Specifically, the min–max normalization method is described as follows:

(13) $$x^{\prime }=\frac{x-\min\left(x\right)}{\max\left(x\right)-\min\left(x\right)}$$

Here, *x* denotes the original feature value, min(*x*) represents the minimum value of feature across the entire dataset, and max*(x)* is the maximum value of the feature across the entire dataset. Following this transformation, all features were rescaled to the range [0,1], ensuring that no single feature dominated the learning process due to differences in scale.

The DL model was first pretrained and then adapted to clinical diagnostics using real clinical data. Based on the interdependence of human physiological indicators, a 1D‐CNN was employed for feature extraction to capture relationships among locally varying features, as illustrated in Figure 2B. This design serves solely for feature modelling and imposes no constraints on the decoder network architecture. By arranging these three scalars sequentially, a one‐dimensional convolution kernel with size = 3 and padding = 1 simultaneously aggregates adjacent features, explicitly learning first‐order and higher‐order local interactions as a structured inductive bias. Formally, for a feature vector $x=[x_{1},\;x_{2},\;x_{3}],$ of length 3, the one‐dimensional convolution response at position i is $y_{i}=w_{-1}\;x_{i-1}+w_{0}x_{i}+w_{+1}x_{i+1}+b$. This captures the local coupling of $(x_{1},\;x_{2},\;x_{3}$) within a single convolution operation, rather than performing independent linear transformations on each dimension. Compared with other feature encoders (such as those employing fully connected networks directly), the convolution mechanism with locally shared parameters more stably emphasises the combinatorial patterns of neighbouring features. This constitutes a core prior for our task, independent of the specific network architecture used for decoding or the input feature dimensions. Moreover, our design extends beyond the three‐dimensional features discussed herein. For inputs exceeding three dimensions, multiscale 1D kernels can similarly extract dependencies. This approach captures multiscale dependencies between local features without constraining the specific architecture of subsequent decoding networks.

Preprocessed data passed through two convolutional layers for local feature extraction, and two fully connected layers for higher‐dimensional features. The output layer applied inverse normalization to yield predicted temperatures.

During model training, hyperparameter selection adheres to the principle of “stable training—enhanced generalization.” The initial learning rate is set to 1 × 10^−^^3^ and employs a cosine annealing learning strategy for dynamic decay. This facilitates rapid convergence in the early stages while progressively reducing the learning rate in later phases to refine the optimization process. The optimizer Adam is selected for its adaptive adjustment of parameter update steps, maintaining training stability across varying gradient magnitudes. The Rectified Linear Unit was used as the activation function. Weights underwent Gaussian random initialization to enhance reproducibility.

To adapt the model for clinical diagnostics, after pretraining, the mapping from the hidden layers to the output layer was fine‐tuned using real clinical data. This ensured that the mapping relationship aligned with the distribution of the clinical data space, while preserving the features learned by the lower layers of the network.

Based on Equation (1) and Pennes’ bio‐heat transfer equation, we assumed that the feature mapping relationship and physiological temperature generation mechanisms remain consistent between healthy individuals and patients. Although numerical values vary significantly under different physiological states, the mathematical form remains consistent. Consequently, we generated a substantial number of physiologically plausible synthetic samples from limited real‐world data of healthy subjects and heat transfer models for model pretraining to learn a general physiological mapping prior. Subsequently, fine‐tuning with real patient data enabled adaptive correction for feature differences under pathological conditions. This strategy ensures the physiological plausibility of model parameters.

### 5.2 Brain Tissue 3D Model Construction

**FIGURE 6:**
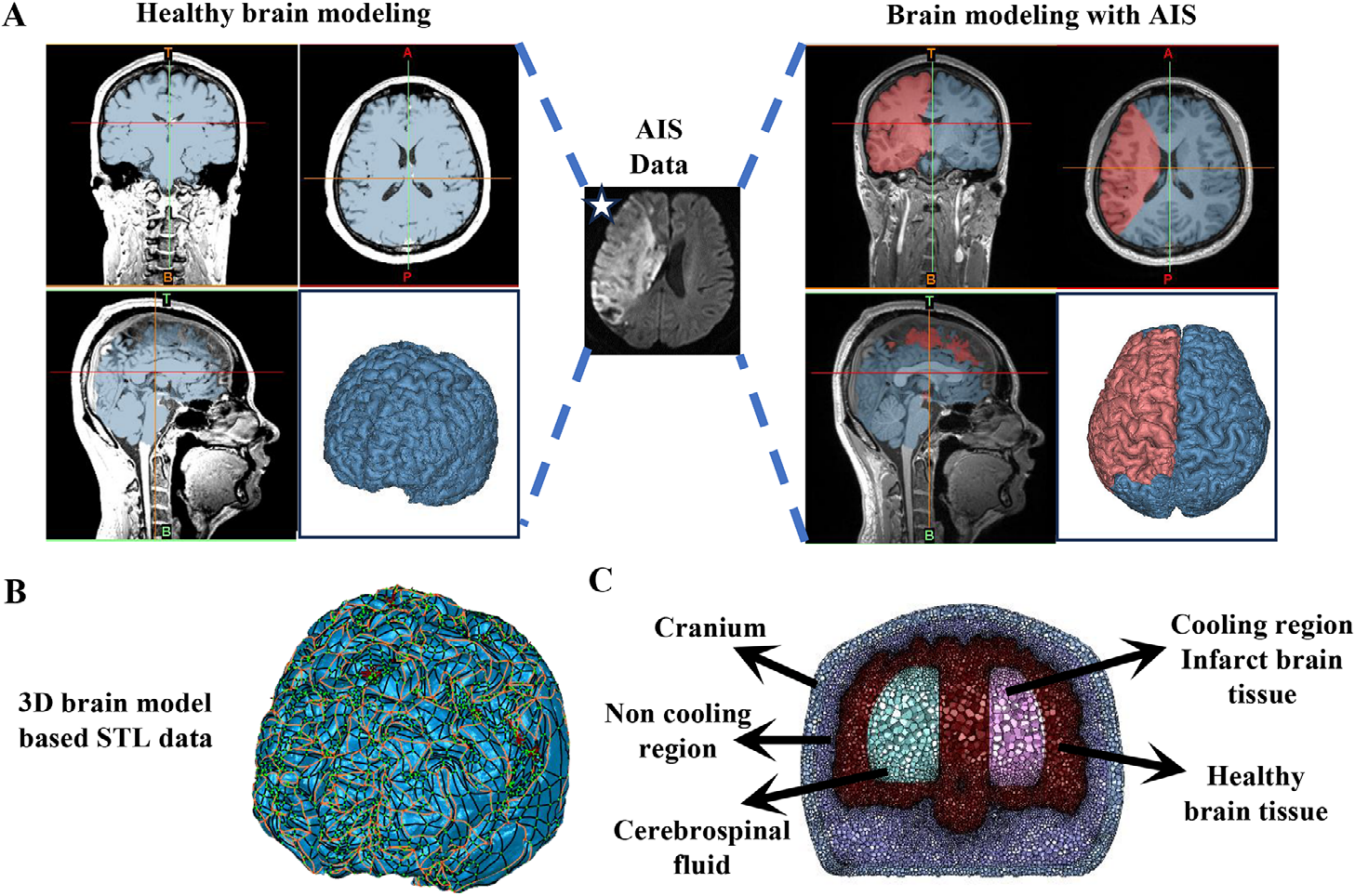
Construction process of the brain tissue three‐dimensional (3D) model and computational imaging. (A) High‐fidelity 3D modeling of healthy brain tissue, ischemic brain tissue, cerebrospinal fluid, and the skull, constructed from magnetic resonance imaging (MRI) data. (B) The 3D brain model based stereolithography (STL) data. (C) The finite element method (FEM) model, including regions of normal‐temperature brain tissue, low‐temperature brain tissue, cerebrospinal fluid, and the skull.

From high‐resolution patient MRI, a 3D model comprising healthy and ischemic brain tissue, cerebrospinal fluid, and the skull was constructed in Mimics Research 21.0 (Materialize) (Figure 6A). The brain tissue and skull were separately isolated and refined using thresholding and segmentation masks to remove irregularities, ensuring a smooth representation. The resulting STL surface file (Figure 6B) was reverse‐engineered in Geomagic Wrap 2021 (3D Systems) into a solid STP model. Surface irregularities were corrected by iterative application of the “sculpting tool,” and the surface model was refined by fitting a grid to ensure uniformity. The brain tissue was segmented into hypothermic and nonhypothermic regions (Figure 6C) to simulate localized cooling interventions.

### 5.3 Computational Imaging Method

The STP format file was imported into SpaceClaim (ANSYS) to define and label the regions corresponding to normal‐temperature brain tissue, low‐temperature brain tissue, cerebrospinal fluid, and the skull. Conjugate coupling was applied to these four regions to enable heat transfer between solid and fluid domains. As shown in Figure 6, an unstructured polyhedral mesh was generated for the intracranial brain tissue model in Fluent Meshing (ANSYS). The mesh size ranged from 0.4 to 4 mm, resulting in 598,860 total mesh elements and a minimum orthogonality quality of 0.23. The energy equation and the Realizable k‐ε turbulence model with enhanced wall treatment were selected for the simulation. Both the brain tissue and cerebrospinal fluid were modeled as incompressible fluids with constant density. The pressure‐based solver was used to couple the pressure and velocity fields through the continuity equation. Velocity‐pressure coupling was solved using the SIMPLEC algorithm, with enhanced wall treatment accounting for pressure gradient effects. Viscosity and density at the interfaces were computed as the arithmetic average of adjacent nodes. The pressure field was discretized using the standard method, while other variables employed a second‐order upwind scheme for improved accuracy.

Temperature data were mapped onto MRI images and linearly interpolated. MRI segmentation was performed using the Image Segment tool in MATLAB (R2017b; The MathWorks) with a flood‐fill algorithm to delineate brain tissue regions and their spatial coordinates. Temperature distributions, generated via linear interpolation in the Fieldtrip toolbox, were projected onto inverted MRI slices to produce thermographic and metabolic maps.

### 5.4 Statistical Analysis

Continuous variables are presented as mean ± standard deviation unless otherwise stated. Model performance was assessed by MSE, RMSE, MAE, Med‐AE, and *R* ^2^. Fivefold cross‐validation was used for model evaluation, and paired two‐tailed *t*‐tests were used for fold‐wise comparisons. Pearson and Spearman correlation analyses were performed to evaluate associations among physiological variables. Absolute errors were descriptively reported for the independent AIS patient tests because of the limited sample size. A two‐sided *p* < 0.05 was considered statistically significant.

## Author Contributions

T.Y., M.J., and Y.H. conceived and designed the study. T.Y., M.J., and H.K. designed the deep‐learning network. Q.Z., Y.W., F.C., X.Z., M.W., and J.Y. contributed to the acquisition and analysis of data. Q.M., M.L., and X.J. coordinated and oversaw the study. T.Y. and M.J. wrote the manuscript with input from all authors. All authors have read and approved the final manuscript.

## Funding

The authors have nothing to report.

## Ethics Statement

This study has been reviewed and approved by the Ethics Committee of Xuanwu Hospital, Capital Medical University, approval number: 2021–053. Informed consent was obtained from all participants involved in the study.

## Conflicts of Interest

The authors declare no conflicts of interest.

## Supporting information




**Figure S1**. Comparison of predicted and measured brain temperature using the proposed noninvasive system.
**Table S1**. The current state of research in China and internationally regarding the application of brain temperature prediction models in analyzing brain temperature regulation and heat transfer mechanisms in neurological disorders.

## Data Availability

The datasets used and analyzed during the current study available from the corresponding author on reasonable request.
