# Supplementary material for: Deep Learning‐Driven Computational Imaging for Noninvasive Monitoring System of Brain Temperature and Metabolism: A Hypothermia Validation for Acute Ischemic Stroke
Source: MedComm (2020). 2026 Aug 5;7(8):e70841. doi: 10.1002/mco2.70841 (PMC13439221; doi:10.1002/mco2.70841)
Supplement: Supplementary file 1 [file MCO2-7-e70841-s001.docx]

**Supplementary Materials**

**Deep Learning-Driven Computational Imaging for Non-Invasive Monitoring System of Brain Temperature and Metabolism: A Hypothermia Validation for Acute Ischemic Stroke**

# Author names and affiliations:

Tianhang Yang^1,2#^, Qihan Zhang^3,4#^, Yilun Huang^5#^, Yuan Wang^4^, Fuzhi Cao^6^, Xin Zhang^7^, Ming Wei^2,8^, Qingfeng Ma^4^, Hongzhi Kuai^9^, Ming Li^3^, Jianzhuo Yan^1*^, Miaowen Jiang^2,3*^, Xunming Ji^2,3,4*^

1 School of Information Science and Technology, Beijing University of Technology, Beijing 100124, China

2 Beijing Institute of Brain Disorders, Capital Medical University, Beijing 100069, China

3 China-America Institute of Neuroscience and Beijing Institute of Geriatrics, Xuanwu Hospital, Capital Medical University, Beijing 100053, China

4 Department of Neurology and Neurosurgery, Xuanwu Hospital, Capital Medical University, Beijing 100053, China

5 School of Instrumentation and Optoelectronic Engineering, Beihang University, Beijing 100083, China

6 School of Engineering Medicine, Beihang University, Beijing 100083, China

7 Brainnetome Center, Laboratory of Brain Atlas and Brain-inspired Intelligence, Institute of Automation, Chinese Academy of Sciences, Beijing 100190, China

8 Tianjin Huanhu Hospital, Tianjin University, Tianjin 300203, China

9 Graduate School of Engineering, Maebashi Institute of Technology, Maebashi 371-0816, Japan

# Corresponding Author: Jianzhuo Yan, Miaowen Jiang and Xunming Ji

Email: [yanjianzhuo@bjut.edu.cn](mailto:yanjianzhuo@bjut.edu.cn); [jiangmiaowen@ccmu.edu.cn](mailto:jiangmiaowen@ccmu.edu.cn); [jixm@ccmu.edu.cn](mailto:jixm@ccmu.edu.cn)

**^#^These authors contributed equally to this work.**

1. **Clinical feasibility evaluation**

The overall clinical dataset of 10 AIS patients remains moderate and is currently focused on a single pathology. The framework's validation also relied on retrospective analyses. The model's performance and generalizability in other neurological diseases have not been evaluated. Future iterations will prioritize expanding both dataset diversity and prospective trials, extending validation to other patient populations to fully assess the clinical utility of the framework. The error distribution of the non-invasive BT prediction system was supplemented using a small number of additional clinical samples. Results indicated a median absolute error (MedAE) of 0.1121℃, mean absolute error (MAE) of 0.1729℃, maximum absolute error (MaxAE) of 0.3819℃, and minimum absolute error (MinAE) of 0.0292℃ between predicted and actual BT measurements. Compared with the original test results from five patients, the error distribution remained broadly consistent overall. No increase in the upper bound of error was observed, while the maximum absolute error showed a slight reduction, as shown in the figure below. These findings indicate that the model's predictive performance remains fundamentally stable following a modest increase in clinical sample size, exhibiting a trend towards slight improvement. Although the current sample size remains limited, these supplementary results provide indirect support for the system's stability in real clinical settings and suggest potential for gradual optimisation as data accumulates.

Figure S1. Comparison of predicted and measured brain temperature using the proposed non-invasive system

2. **Supplementary Derivation Process for the Brain Temperature Prediction Model**

**We have supplemented the theoretical basis of the brain temperature prediction model with detailed derivations and explanations:“**The theoretical foundation model for brain temperature prediction is: the Pennes model of biological heat transfer is founded upon **Fourier's law** and **tissue energy balance**, incorporating a pivotal and revolutionary simplifying assumption regarding the effects of blood perfusion. **Originating from the classical heat conduction equation,** the transient heat transfer process in homogeneous solids is described by the classical Fourier heat conduction equation:

$$\rho c\frac{\partial T}{\partial t}=\nabla\cdot(k\nabla T)+\dot{q}_{met}+\dot{q}_{ext}$$

Where: T denotes the temperature (°C or K); t represents the time (s); ρ is the tissue density (kg/m³); c is the tissue specific heat capacity (J/kg·K); k denotes the thermal conductivity of the tissue (W/m·K); $\dot{q}_{met}$ refers to the metabolic heat production rate (W/m³); $\dot{q}_{ext}$ denotes the heat generation rate from external sources such as laser or microwave irradiation (W/m³); and $\nabla\cdot(k\nabla T)$ describes the heat diffusion (conduction) arising from spatial temperature gradients.

**The issue with this equation lies in its failure to account for heat exchange brought about by flowing blood. The model's core contribution lies in its blood perfusion term,** Pennes did not attempt to precisely describe blood flow and heat exchange within complex vascular networks (a task that remains exceedingly difficult both then and now). Instead, he proposed a macroscopic, lumped-parameter hypothesis: **the bioheat equation**:

$$\rho_{t}c_{t}\frac{\partial T}{\partial t}=\nabla\cdot\left( k_{t}\nabla T \right)+\omega_{b}\rho_{b}c_{b}\left( T_{a}-T_{v} \right)+\dot{q}_{met}+\dot{q}_{ext}$$

The newly introduced term $\omega_{b}\rho_{b}c_{b}\left( T_{a}-T_{v} \right)$ represents **the blood perfusion component**, which is the **core contribution of the Pennes model**. In this formulation, $\omega_{b}$ denotes **the blood perfusion rate**, with units of m³/s (often expressed as kg/(m³·s) or 1/s), and reflects the mass of blood passing through a unit volume of tissue per unit time. The quantity $\rho_{b}c_{b}$ corresponds to the volumetric heat **capacity of blood** (J/m³·K), while the term $\left( T_{a}-T_{v} \right)$ denotes the **temperature difference between arterial and venous blood at the local capillary bed.** This perfusion term captures the convective heat exchange between circulating blood and surrounding tissue and **embodies the essential physiological assumption underlying the Pennes model**.

**The key assumption of the model is the** **“thermal equilibrium assumption”**. The most fundamental and subsequently most debated assumption of the Pennes model is that, **at the capillary level, arterial blood and the surrounding tissue can instantly achieve thermal equilibrium. The temperature of venous blood leaving the capillary equals the temperature T of the tissue at that point. That is:** $T_{v}=T$. Substituting this assumption into the blood perfusion term yields the final form of Pennes' biothermal equation:

$$\rho_{t}c_{t}\frac{\partial T}{\partial t}=\nabla\cdot(k_{t}\nabla T)+\omega_{b}\rho_{b}c_{b}(T_{a}-T)+\dot{q}_{met}+\dot{q}_{ext}.$$

3. **Details of the three-dimensional finite element analysis model**

**we have added a more detailed description of the 3D model construction, boundary condition setup, and numerical solution procedures.** Specifically, utilising MRI data in the Digital Imaging and Communications in Medicine (DICOM) format, the three-dimensional geometric structures of brain tissue and cerebral vasculature were reconstructed via surface modelling using Mimics (Materialise NV, Leuven, Belgium) and Geomagic Wrap (Geomagic, North Carolina, USA). Subsequently, ANSYS ICEM CFD (ANSYS, Inc., USA) was employed for mesh generation and computational domain creation. Blood flow was modelled as an incompressible viscous Newtonian fluid, accounting for compressibility effects at vessel walls and thermal conduction. Given the complex vascular geometry, unstructured tetrahedral elements were used for domain discretisation, with finer meshing near vessel walls and narrow passages to capture local flow variations. Blood flow was treated as a perfusion term, with a nominal vessel wall thickness of approximately 2.0 mm and no-slip boundary conditions applied. Initial conditions were set to zero, with inlet boundaries corresponding to the internal carotid or vertebral arteries and outlet boundaries to distal cerebral arteries and cold perfusion solution temperature. Convergence criteria were set to a root mean square (RMS) relative residual target of 1.0×10^-^⁵, with blood flow assumed laminar.

4. **Further discussion on clinical application value**

**we have supplemented the potential value of 3D brain temperature imaging and brain metabolic heat production imaging results in clinical decision-making.** This model may serve as a virtual sensor, enabling dynamic and individualised prediction of brain temperature through the fusion of multimodal data (e.g., frontal skin temperature, heart rate variability, electroencephalogram, body core temperature, and even invasive monitoring metrics) with artificial intelligence modelling.Multimodal data fusion and computational AI modelling, integrating the aforementioned non-invasive signals (e.g., frontal skin temperature, heart rate, other physiological parameters), and even incorporating limited invasive monitoring data, to utilise artificial intelligence models for more accurate brain temperature prediction.’ This represents a promising developmental direction, primarily enabling: (1) Multi-source inputs: The model incorporates not only frontal lobe temperature but also integrates heart rate variability, electroencephalogram, intracranial pressure (where invasive monitoring is available), core body temperature, and even imaging features. These parameters collectively form a ‘physiological state panorama’ influencing brain temperature. (2) AI models as ‘virtual sensors’: Deep learning algorithms learn deep, non-linear mapping relationships between these complex physiological signals and gold-standard brain temperature (e.g., intraoperative or invasive monitoring data) from vast historical datasets. (3) Achieving dynamic and personalised prediction: Mature models can dynamically estimate core brain temperature—which cannot be directly measured—based on real-time physiological stream data. Through continuous learning, they adapt to individual patient variations, ultimately enabling highly accurate, fully automated, bedside non-invasive brain temperature monitoring. This provides critical decision support for precision brain protection.

5. **Supplemental Table**

Table S1. The current state of research in China and internationally regarding the application of brain temperature prediction models in analyzing brain temperature regulation and heat transfer mechanisms in neurological disorders.

| Nervous system disease | Clinical application | Research content: Innovation (1), Limitation (2) and experimental verification (3) | References |
| --- | --- | --- | --- |
| Ischemic hypoxic brain injury | Evaluation and mechanism of brain temperature in therapeutic hypothermia intervention in hypoxic-ischemic encephalopathy | (1) A numerical model was developed to study the effects of hypothermia on cerebral blood flow and metabolism. The model predicted that during hypothermia treatment, cerebral metabolism decreased by 5.1%, cerebral blood flow decreased by 1.1%, and cerebral tissue oxygenation saturation increased by 2.3% | Britain (2020)^[1]^ |
|  |  | (2) Replace brain temperature with body temperature |  |
|  |  | (3) Numerical validation, no MRI/CT image evaluation |  |
| Ischemic hypoxic brain injury | Measurement and mechanism of brain temperature during neuroprotective period of deep hypothermic cardiopulmonary bypass | Non-invasive frequency-domain diffusion spectroscopy (FD-DOS) and diffusion correlation spectroscopy (DCS) were utilized to directly measure the cerebral oxygen metabolic rate (CMRO₂), and the temperature dependence of CMRO₂ was proposed | USA (2018)^[2]^ |
|  |  | (2) Use ear and nose temperature instead of brain temperature |  |
|  |  | (3) Animal experiments verified, no MRI/CT image evaluation |  |
| Ischemic hypoxic brain injury | Evaluation and mechanism of brain temperature in the treatment of cardiac arrest with therapeutic hypothermia | (1) Using the anatomical features of the skull and carotid artery from CT images, a finite element analysis model was developed to predict and evaluate brain temperature distribution under low temperature conditions | USA (2018)^[3]^ |
|  |  | (2) Without accounting for the characteristics of the cerebral vascular network structure, the continuum model cannot accurately predict temperature changes near discrete blood vessels |  |
|  |  | (3) In vitro experiments confirmed that CT imaging assisted in the modeling of the skull and carotid artery |  |
| Ischemic hypoxic brain injury | Noninvasive estimation of brain temperature and monitoring of therapeutic hypothermia in target temperature management | The DSM algorithm for non-invasive sensor monitoring was applied to brain temperature monitoring, and a brain temperature estimation algorithm was proposed. By incorporating measured values, the accuracy of the algorithm was enhanced | USA (2019)^[4]^ |
|  |  | (2) The traditional DSM temperature estimation model did not adequately account for heat transfer in biological tissues |  |
|  |  | (3) Animal experiments verified, no MRI/CT image evaluation |  |
| Ischemic hypoxic brain injury | Hemodynamic regulation of brain temperature during hypothermia | (1) 1D arterial and venous heat transfer model was developed using a 3D fluid-porous domain coupling approach. The Vascular Porous (VaPor) model incorporates the vascular system extracted from MRI data, accounting for heat generated by metabolism and the cerebral blood flow energy equation | Britain (2018)^[5]^ |
|  |  | (2) The coupling analysis of 3D blood flow temperature and heat transfer within the flow field is insufficient |  |
|  |  | (3) Numerical validation was performed, guided by MRI/CT imaging for cerebrovascular modeling |  |
| Ischemic hypoxic brain injury | Evaluation of brain temperature during hypothermia therapy for neonatal ischemic hypoxic encephalopathy | (1) A brain temperature prediction model was developed based on the inverse analysis method using the standard least squares approach. The model was analyzed, and its results were compared with measured data obtained during actual treatment | Poland (2016)^[6]^ |
|  |  | (2) There is a lack of hemodynamic modeling analysis for the vascular network |  |
|  |  | (3) Clinical trial verification, MRI/CT image evaluation |  |
| Ischemic stroke | Brain tissue heat transfer and the mechanism of hypothermia-induced neuroprotection during intracranial targeted lesion cooling | (1) A multi-physical brain temperature calculation model was proposed, which can accurately predict the brain response to focal cooling. The magnetic resonance thermometer was used to monitor the brain temperature during local cerebral cooling | USA (2022)^[7]^ |
|  |  | (2) The dispersion of brain tissue was not considered, and the effect of cerebral hemodynamics on brain temperature was ignored |  |
|  |  | (3) Clinical validation, MRI/CT imaging evaluation |  |
| Ischemic stroke | Evaluation of brain temperature and the hemodynamic mechanisms of selective brain hypothermia for ischemic stroke | (1) A biophysical model based on the first principle of conservation of energy and mass was proposed. The model used magnetic resonance imaging data from individual brain tissue and blood vessel structure to improve the precision of personalized brain tissue heat transfer | USA (2021)^[8]^ |
|  |  | (2) Hemodynamic spatial heat flow coupling analysis without real vascular network model |  |
|  |  | (3) Clinical validation, MRI/CT imaging evaluation |  |
| Ischemic stroke | Evaluation of brain temperature and the hemodynamic mechanisms of selective brain hypothermia for ischemic MCA-M1 stroke | (1) A model for predicting brain temperature with spatial resolution and distinguishing between white and gray matter is proposed, enabling for the first time a detailed anatomical structure of brain circulation that can be personalized according to real patient anatomy | Germany (2021)^[9]^ |
|  |  | (2) The cerebral vascular network equivalent model was adopted, and the model was not based on CT/MRI data |  |
|  |  | (3) Numerical validation, no MRI/CT image evaluation |  |
| Ischemic stroke | Evaluation and mechanism of brain temperature in mild hypothermia neuroprotection | (1) An analytical model for brain hypothermia heat transfer, based on the brain’s structure and heat transfer mechanism, has been proposed. The analytical solution of the Pennes bioheat transfer equation was derived in a one-dimensional spherical coordinate system (radial) using the Laplace transform method | China (2016)^[10]^ |
|  |  | (2) The brain is reduced to a two-layer structure composed of scalp and brain tissue |  |
|  |  | (3) Animal studies confirmed, no MRI/CT image evaluation |  |
| Ischemic stroke | Evaluation and mechanisms of intravascular and ice cap hypothermia combined with neuroprotection of midbrain temperature | (1) A feedback method for local brain temperature control was proposed, incorporating blood dilution and volume load modeling to facilitate convective heat transfer through jugular reflux and the Circle of Willis. | USA (2008)^[11]^ |
|  |  | (2) The intracranial anatomy was simplified to a basic hemispherical model. As a result, the continuum model was unable to accurately predict temperature changes near discrete blood vessels. Additionally, the effects of cerebral blood flow on local temperature and hematocrit were not accounted for. |  |
|  |  | (3) Numerical validation, no MRI/CT image evaluation |  |
| Ischemic stroke | Evaluation and mechanisms of midbrain temperature in intravascular hypothermia neuroprotection | (1) A method combining a vascular model and a continuum model has been proposed to predict the temperature field in the neck and brain | USA (2007)^[12]^ |
|  |  | (2) The transient variation of heat flux and boundary temperature as a function of spatial position was not considered, and the continuum model was unable to predict temperature variations near discrete blood vessels |  |
|  |  | (3) Numerical validation, no MRI/CT image evaluation |  |
| Hemorrhagic stroke | Evaluation and mechanisms of intravascular and ice cap hypothermia combined with neuroprotection on midbrain temperature regulation | (1) Models incorporating MRI data, thermal conductivity, metabolism, and blood flow were applied to evaluate various hypothermia conditions in patients with severe subarachnoid hemorrhage | Zurich (2009)^[13]^ |
|  |  | (2) Transient changes in local cerebral blood flow, cerebral metabolism, and perfused arterial blood temperature were not considered |  |
|  |  | (3) Clinical validation, MRI/CT imaging evaluation |  |
| Brain tumor | Measurement and evaluation of brain temperature during laser ablation therapy for brain tumors | (1) The significance of temperature-dependent characteristics, non-Fourier behavior, and the microvascular perfusion effect in brain tissue was highlighted | Canada (2024)^[14]^ |
|  |  | (2) Tumors, brain tissue, and blood areas were not distinguished |  |
|  |  | (3) No MRI/CT imaging evaluation |  |
| Brain tumor | Estimation of brain tumor temperature distribution across various sizes and depths | (1) A parallel GPU algorithm for monitoring brain tumor temperature (with a maximum temperature rise of 0.32°C) was proposed to analyze the correlation between brain tumor temperature, metabolic heat production, and blood perfusion rate | Morocco (2018)^[15]^ |
|  |  | (2) The relative error between the exact and estimated values of the blood perfusion rate are significant |  |
|  |  | (3) Numerical validation, no MRI/CT image evaluation |  |
| Brain tumor | Brain temperature measurement and surgical planning for laser ablation therapy of brain tumors | (1) A cross-validation analysis was proposed to evaluate the temperature distribution in brain tissue during magnetic resonance-guided laser-induced heat treatment for focal lesions. Additionally, a closed-form algorithm was developed to predict steady-state heat transfer using the linear superposition of analytic point source heating functions | USA (2015)^[16]^ |
|  |  | (2) The spectral element discrete method, which only considers the transient Pennes bioheat transfer equation, fails to account for the dynamic characteristics of the blood perfusion term |  |
|  |  | (3) Clinical validation, MRI/CT imaging evaluation |  |
| Brain tumor | Thermal image monitoring and brain temperature evaluation of brain tumors during laser thermal ablation guided by nuclear magnetic resonance | (1) The Pennes bioheat transfer model, combined with the finite element parameter inversion method, was utilized to estimate optical parameters and evaluate the mechanism of brain temperature inversion during magnetic resonance temperature imaging | USA (2013)^[17]^ |
|  |  | (2) The metabolic components of brain tissue were ignored |  |
|  |  | (3) It was verified by animal experiments and evaluated by MRI/CT images |  |
| Brain tumor | Prediction of brain temperature distribution in a noninvasively hyperthermia system for brain tumors | (1) A new non-invasive hyperthermia system for brain tumors was proposed, and the mathematical model of brain tissue temperature was decoupled using the finite-difference time-domain method | Egypt (2012)^[18]^ |
|  |  | (2) The Pennes bioheat transfer equation is limited in calculating temperature rise as it neglects the vasculature within brain tissue |  |
|  |  | (3) Numerical validation, no MRI/CT image evaluation |  |
| Cerebral glioblastoma | Evaluation of the effect of hyperthermia on glioblastoma multiforme through brain tissue temperature analysis | (1) The bioheat transfer equations of Maxwell and Pennes were used to numerically verify the temperature distribution of glioblastoma multiforme, and the corresponding parameters were designed | Türkiye (2022)^[19]^ |
|  |  | (2) There is a lack of thermodynamic analysis based on realistic 3D realistic models of brain tissue |  |
|  |  | (3) Numerical validation, no MRI/CT image evaluation |  |
| Neurological intensive care | Evaluation of brain temperature during active fluid warming-assisted rewarming in hypothermia therapy | (1) A steady-state, time- and temperature-dependent thermodynamic model was proposed to evaluate the effects of intravenous infusion of temperature-controlled cold liquid | USA (2012)^[20]^ |
|  |  | (2) The model ignores thermal radiation, heat conduction, and evaporation from biological tissues |  |
|  |  | (3) Clinical studies verified, no MRI/CT image evaluation |  |
| Neurological intensive care | Evaluation of cerebrospinal fluid and spinal cord temperature following simple cord injury induced by a simple cooling pad | (1) Using the Pennes biothermal equation and the finite element method, the temperature distribution of the spinal cord and cerebrospinal fluid during 30 minutes of cooling in patients with spinal cord injury was analyzed. The study revealed that the reduction in spinal cord temperature was influenced by blood perfusion rate, cooling temperature, and cerebrospinal fluid flow. | USA (2010)^[21]^ |
|  |  | (2) Thermodynamic analysis of the coupling between the spinal cord and cerebrospinal fluid was not conducted using realistic models |  |
|  |  | (3) Numerical validation, no MRI/CT image evaluation |  |

**References**

[1] J. Russell-Buckland and I. Tachtsidis, “Developing a Model to Simulate the Effect of Hypothermia on Cerebral Blood Flow and Metabolism,” Advances in Experimental Medicine and Biology 1232 (2020): 299–306.

[2] T. S. Ko, C. D. Mavroudis, W. B. Baker, et al., “Non-invasive Optical Neuromonitoring of the Temperature-dependence of Cerebral Oxygen Metabolism During Deep Hypothermic Cardiopulmonary Bypass in Neonatal Swine,” Journal of Cerebral Blood Flow & Metabolism 40, no. 1 (2018): 187–203.

[3] H. Li, R. K. Chen, Y. Tang, W. Meurer, and A. J. Shih, “An Experimental Study and Finite Element Modeling of Head and Neck Cooling for Brain Hypothermia,” Journal of Thermal Biology 71 (2018): 99–111.

[4] D. S. Sung, S. Y. Sim, H. W. Jin, et al., “Validation of Non-invasive Brain Temperature Estimation Models During Swine Therapeutic Hypothermia,” Physiological Measurement 40, no. 2 (2019): 025004.

[5] S. Blowers, I. Marshall, M. Thrippleton, et al., “How Does Blood Regulate Cerebral Temperatures During Hypothermia?,” Scientific Reports 8, no. 1 (2018): 7877.

[6] J. E. Laszczyk, P. Božidar Šarler, and A. J. Nowak, “Computational Modelling of Neonate’s Brain Cooling,” International Journal of Numerical Methods for Heat & Fluid Flow 26, no. 2 (2016): 571–590.

[7] T. S. Baker, A. L. Zannou, D. Cruz, et al., “Development and Clinical Validation of a Finite Element Method Model Mapping Focal Intracranial Cooling,” IEEE Transactions on Neural Systems and Rehabilitation Engineering 30 (2022): 2168–2174.

[8] D. Sung, P. A. Kottke, B. B. Risk, et al., “Personalized Predictions and Non-invasive Imaging of Human Brain Temperature,” Communications Physics 4, no. 1 (2021): 68.

[9] Y. Lutz, T. Meiner, L. Krames, et al., “Selective Brain Hypothermia for Ischemic MCA-M1 Stroke: Influence of Cerebral Arterial Circulation in a 3D Brain Temperature Model,” IEEE Transactions on Biomedical Engineering 68, no. 2 (2021): 404–415.

[10] W. Ma, W. Liu, and M. Li, “Analytical Heat Transfer Model for Targeted Brain Hypothermia,” International Journal of Thermal Sciences 100 (2016): 66–74.

[11] M. A. Neimark, A.-A. Konstas, J. H. Choi, A. F. Laine, and J. Pile-Spellman, “Brain Cooling Maintenance With Cooling Cap Following Induction With Intracarotid Cold Saline Infusion: A Quantitative Model,” Journal of Theoretical Biology 253, no. 2 (2008): 333–344.

[12] Y. Wang and L. Zhu, “Targeted Brain Hypothermia Induced by an Interstitial Cooling Device in Human Neck: Theoretical Analyses,” European Journal of Applied Physiology 101, no. 1 (2007): 31–40.

[13] E. Keller, R. Mudra, C. Gugl, M. Seule, S. Mink, and J. Fröhlich, “Theoretical Evaluations of Therapeutic Systemic and Local Cerebral Hypothermia,” Journal of Neuroscience Methods 178, no. 2 (2009): 345–349.

[14] S. Singh, L. Bianchi, S. Korganbayev, P. Namakshenas, R. Melnik, and P. Saccomandi, “Non-Fourier Bioheat Transfer Analysis in Brain Tissue During Interstitial Laser Ablation: Analysis of Multiple Influential Factors,” Annals of Biomedical Engineering 52, no. 4 (2024): 967–981.

[15] A. Bousselham, O. Bouattane, M. Youssfi, and A. Raihani, “3D Brain Tumor Localization and Parameter Estimation Using Thermographic Approach on GPU,” Journal of Thermal Biology 71 (2018): 52–61.

[16] S. J. Fahrenholtz, T. Y. Moon, M. Franco, et al., “A Model Evaluation Study for Treatment Planning of Laser-induced Thermal Therapy,” International Journal of Hyperthermia 31, no. 7 (2015): 705–714.

[17] D. Fuentes, A. Elliott, J. S. Weinberg, A. Shetty, J. D. Hazle, and R. J. Stafford, “An Inverse Problem Approach to Recovery of In Vivo Nanoparticle Concentrations From Thermal Image Monitoring of MR-guided Laser Induced Thermal Therapy,” Annals of Biomedical Engineering 41, no. 1 (2013): 100–111.

[18] S. M. Yacoob and N. S. Hassan, “FDTD Analysis of a Noninvasive Hyperthermia System for Brain Tumors,” Biomedical Engineering Online 11 (2012): 47.

[19] A. Sağıroğlu, İ. Karagöz, Ö. Ö. Özcan, T. T. Ergüzel, M. Karahan, and N. Tarhan, “Evaluation of the Glioblastoma Multiforme Treatment With Hyperthermia Using the Finite Element Method,” Numerical Heat Transfer, Part A: Applications 83, no. 11 (2022): 1239–1260.

[20] E. R. Barthel and J. R. Pierce, “Steady-state and Time-dependent Thermodynamic Modeling of the Effect of Intravenous Infusion of Warm and Cold Fluids,” Journal of Trauma and Acute Care Surgery 72, no. 6 (2012): 1590–1600.

[21] K. D. Smith and L. Zhu, “Theoretical Evaluation of a Simple Cooling Pad for Inducing Hypothermia in the Spinal Cord Following Traumatic Injury,” Medical & Biological Engineering & Computing 48, no. 2 (2010): 167–175.
